# The effects of *Rosa foetida* extract along with self-care education on primary dysmenorrhea: study protocol for a randomized clinical trial

**DOI:** 10.1186/s13063-022-06583-4

**Published:** 2022-08-09

**Authors:** Fatemeh Shabani, Shabnam Omidvar, Parvin Sajadi Kaboudi, Hajar Pasha, Soraya Khafri, Hossein Najafzadehvarzi, Fatemeh Nasiri Amiri, Mahbobeh Faramarzi, Zinatossadat Bouzari

**Affiliations:** 1 Student Research Committee, Babol University of Medical Sciences, Babol, Islamic Republic of Iran; 2 Infertility and Health Reproductive Research Center, Health Research Institute, Babol University of Medical Sciences, Babol, Islamic Republic of Iran; 3 Social Determinants of Health Research Center, Health Research Institute, Babol University of Medical Sciences, Babol, Islamic Republic of Iran; 4 Department of Statistic and Epidemiology, Health Research Institute, Babol University of Medical Sciences, Babol, Islamic Republic of Iran; 5 Cellular and Molecular Biology Research Center, Health research Institute, Babol University of Medical Sciences, Babol, Islamic Republic of Iran

**Keywords:** Dysmenorrhea, Self-caring, Medicinal plants, Clinical protocol

## Abstract

**Background:**

Dysmenorrhea is one of the most common disorders among young women. Medicinal herbs are one of the alternative methods for the treatment of dysmenorrhea. This study will investigate the effect of *Rosa foetida* extract, along with self-care behavior education on primary dysmenorrhea among female students of Babol University of medical sciences.

**Methods/design:**

A randomized clinical trial will be performed on single students, aged 18 to 24 years. The research samples will be divided into three groups. The students will receive self-care behavior education on dysmenorrhea. Following the education, two of the groups will receive *Rosa foetida* extract capsules and placebo capsules in two consecutive cycles every 8 h for two successive days, respectively. The capsules will have similar physical appearance. The third group will not receive any medication. Data will be collected through demographic characteristic questionnaire, visual analog scale, dysmenorrhea self-care behaviors scale questionnaire, pictorial chart, and menstrual distress scale questionnaire. In order to determine and compare the effect of pharmacological and educational interventions on the severity of dysmenorrhea in groups, an ANOVA analysis of variance test with repeated measures will be used by SPSS software version 22.

**Discussion:**

The results will show the effects of *Rosa foetida* extract along with self-care behavior education on primary dysmenorrhea, and beneficial effects that may be found in the trial of this plant may be of use for women with the same problem.

**Ethics and dissemination:**

The study is approved by the Ethics Committee of Babol University of Medical Sciences (IR.MUBABOL.REC.1397.059).

**Trial registration:**

IRCT 20190318043086N1. Registered on 14 June 2019.

## Background

Dysmenorrhea or painful menstruation is referred to as the painful periods which occur before and during menstruation. Dysmenorrhea is divided into two types: primary and secondary [1]. It is reported that 25 to 85% of women in reproductive age suffer from dysmenorrhea [2]. The most recursive symptom of dysmenorrhea is menstrual cramps which happens due to the release of prostaglandins during menstruation. Other symptoms include nausea, vomiting, fatigue, headache, and dizziness, which may lead to 1 to 3 days of unproductivity [3, 4]. Therefore, dysmenorrhea has always been in the spotlight, in social and economical terms, being the most frequent reason behind women’s absence from school or work [5]. Dysmenorrhea causes loss of 600 million work hours and 2 billion dollars, annually in the USA [6]. The same can be said about Japan where dysmenorrhea causes a loss of over 4.2 billion dollars, every year [7]. Women play an important role in every society, be it in employment or their role in families. This causes dysmenorrhea to be one of the most important problems, being researched worldwide [8, 9].

The methods for the management of dysmenorrhea include medicinal treatments and non-medicinal treatments through supplementary and alternative treatment. The most frequently used medications for countering dysmenorrhea include anti-prostaglandin synthesis medicines and non-steroidal anti-inflammatory medicines such as Gelofen, Novafen, naproxen, mefenamic acid, celecoxib, etc. [10]. Many complementary methods have also been proposed, including transcutaneous electrical nerve stimulation (TENS) [11]; exercise [12]; yoga [13]; massage [14, 15]; acupressure [16]; aromatherapy [17, 18]; herbs such as dill, cumin, and ginger [19]; and placebos [20]. These treatments also proved to be effective in soothing dysmenorrhea.

Persian yellow rose, also known as *Rosa foetida*, is an edible rose. It belongs to the Rosacea family. The chemicals in *Rosa foetida* are similar to the ones found in a typical rose. According to clinical studies and evidences presented by the Australian Gene Technology Regulator, roses and their products were reported to be non-poisonous in all forms [21, 22]. Traditional treatments also utilize red roses to treat chest pain, stomachache, blood flow, and digestive disorders [23, 24].

Modern researches on *Rosa damascena* report the anti-microbial, anti-bacterial, anti-inflammation, pain reduction, antioxidant, cancer preventive, anti-viral, anti-epilepsy, anti-depressant, relaxing, and tranquilizing effects of *Rosa foetida* [24].

Iran is one of the countries where rose and specially *Rosa foetida* are cultivated. Western and Central regions of Iran, as well as some parts in the South, are reported to offer rose cultivation [25]. Moreover, there has been no clinical research on the effects of *Rosa foetida* on dysmenorrhea. Hence, we have decided to do this study. The main objective is to investigate the effect of *Rosa foetida* extract along with self-care behavior education on primary dysmenorrhea, and secondary objectives are to compare the effect of *Rosa foetida* along with self-care behavior with placebo and self-care behavior education on menstrual bleeding as well as menstrual distress.

### Research aims

The study aims are to investigate the effect of *Rosa Foetida* extract, along with self-care behavior education on menstrual pain intensity, menstrual distress, and blood flow during menstruation.

## Methods

### Objectives

Our objectives are (1) to compare the severity of menstrual pain before and after intervention among three groups (*Rosa foetida*, placebo, and self-care); (2) to compare menstrual distress before and after intervention among three groups; and (3) to compare menstrual bleeding severity before and after intervention among three groups.

### Design and setting

**Fig. 1:**
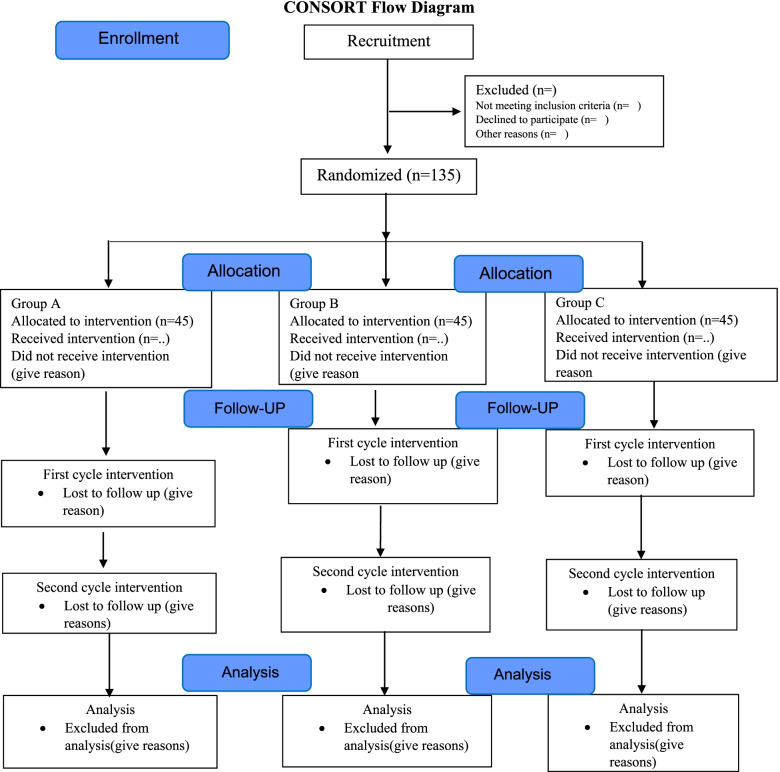
Consolidated Standards of Reporting Trials (CONSORT) flow diagram

This study is a randomized clinical trial that will be performed on students residing at the dormitory of Babol University of Medical Sciences, Babol, Iran. The study design is in accordance with the Consolidated Standards of Reporting Trials (CONSORT) (Fig. 1). The study was approved by Ethics Committee of Babol University of Medical Sciences (IR.MUBABOL.REC.1397.O59), and the study protocol was recorded in the Iranian Registry of Clinical Trial (IRCT 20190318043086N1).

### Participants: eligibility

The interested students will be invited to participate in this study and will be given visual analog scale (VAS) for assessing dysmenorrhea, and those with pain scale equal or higher than 5 will be considered as participants for the study. In addition to that, consent form also will be filled out by the participants.

#### Inclusion criteria

Inclusion criteria are single (unmarried) girls with clinical symptoms of primary dysmenorrhea with pain score (5 or more) based on the visual analogue cale (VAS), who experienced dysmenorrhea in at least 3 of their recent 6 menstruations, aged 18 to 24 with body mass index ranging from 19 to 30 and regular periods, wherein the duration between two consecutive menstruations was ranged from 21 to 35 days. The duration of each bleeding would last 3 to 7 days.

#### Exclusion criteria

Exclusion criteria are any irregularities in the past 3 months, use of non-steroidal inflammatory medicines and anti-prostaglandins in the week before period, and also use of hormonal medication and contraceptive pills in the past 3 months and history of abdominal or pelvic surgery, known allergy to herbs, taking supplements and vitamins, and history of a prominent physical and psychological problems.

The drop-out criteria:

- The diagnosis of gynecological disease during the study by a gynecologist
- Severe stressful events during the study
- Unwilling to continue being as a participant in clinical trials

### Assignment of interventions: allocation

#### Sequence generation

Eligible subjects will be randomly assigned to three groups by block-randomized allocation method with 6 blocks. A randomization will be made by using a website program (http:// random.org). The table will be managed by an individual who is not included in the research team of the trial.

#### Concealment mechanism

After completing a baseline evaluation and determination of eligible participants, all the participants who agree to participate and meet the inclusion criteria will be randomized. The numbers, as generated by the mentioned website, will be given to the researcher who will be in the dark regarding what each intervention envelope included. An independent investigator will provide the random sequence within opaque, sealed envelopes for allocation concealment. The group numbers and the participants will be tracked by another researcher.

#### Implementation

After obtaining permission from the human ethics committee of Babol University of Medical Sciences then registering at the Iranian Clinical Trial Registration Center (IRCT) and obtaining required permissions from university authorities, the researcher will attend at the female dormitory of Babol University of Medical Sciences to invite interested participants for this study. The researcher will provide the information regarding the purpose of the study, kind of intervention, and confidentiality of the information and data. Then participants will be requested to sign the written consent form before enrolling in the study. Moreover, the participants will be ensured that they can leave the study at any stage and they also are allowed to use palliative treatments whenever they are not willing to continue to participate or when they feel need.

A colleague will generate the allocation sequence by using the randomization program. Then, the researcher will get informed consent from the subjects and the colleague will assign the participant to the determined group. The investigator and other related investigators will be blinded to perform proper management.

### Measures

In the research, five instruments will be used for data collection:

1. The demographic characteristic questionnaire will be used to collect demographic and reproductive characteristics, including age, menarche age, menstrual cycle length, menstrual bleeding period, and pain killer use history. The questionnaire has been developed based on objectives of the study and the validity will be examined by the content validity method.
2. Visual analogue scale (VAS). Validity and reliability of the VAS is proven to possess an alpha Cronbach of 0.85 in studies by Bani et al. and Williamson et al. [26, 27].
3. Menstrual Distress Questionnaire (MDQ). The reliability of the Persian version of the MDQ is proven to be 0.93 [28].
4. Dysmenorrhea Self-Care Scale (DSCS) is an edited version of a research instrument proposed by Ching et al. and was validated through content method, in a study by Kabirian et al. In addition to that, the reliability of this method was proven and the alpha Cronbach was reported to be 0.78 [29, 30].
5. Pictorial chart is a self-administered pictorial chart which, in addition to record the number of sanitary pads and tampons used, also takes into account the degree to which individual items are soiled with blood, passage of blood clots, and episodes of flooding. Pictorial chart is also proven to be reliable and valid with an alpha Cronbach 0.84 [31, 32].

#### Primary outcome

The primary outcome is menstrual pain intensity.

#### Secondary outcome

Secondary outcomes are menstrual distress, menstrual blood flow, and the need for pain killer during menstruation.

#### Side effects

The number of side effects experienced by each subject and total number of all the side effects will be summarized for each group.

### Study procedures

#### Extract preparation

In the first step, dried *Rosa foetida* petals will be ground to a fine powder. Then, the powder will be added to a decanter along with 70% ethanol solution for 3–4 h. The solution will be distilled after 24 h. Distillation will be repeated three times. The resultant extract will be added to a rotary device to maximize the concentration of the extract. Finally, the extract will be prepared in the form of a powder which will be encapsulated.

#### Dysmenorrhea self-care education

The training program will be held in four sessions for all the eligible participants in the study. The first and second sessions of the educational content will include anatomy and physiology of menstruation, and the third and fourth sessions of the educational content will include exercise and nutrition.

### Interventions

Group A will receive capsules with 200 mg of *Rosa foetida* extract to be taken three times a day during menstruation days, in addition to self-care instructions. Group B will received capsules with 200 mg of starch (as the placebo group), in addition to self-care instructions. Group C will receive only self-care instructions.

An assistant will code the capsules and seal them in an envelope; then, the envelopes will be given to the researcher to pass to the participants. Each participant will fill out the Pain VAS, Menstrual Distress Questionnaire (MDQ), and Dysmenorrhea Self-Care Scale (DSCS), as well as the pictorial chart in a menstrual cycle before intervention.

Intervention will be done on the first 2 days of two consecutive cycles, and the participants will be requested to take a capsule and then record their pain on the VAS, 1, 2, 4, 8, 12, 24, and 48 h after the start of intervention. In addition to that, the participants will be requested to fill out the MDQ and DSCS at the end of the second day of interventions.

#### Sample size

In order to calculate a sample size, the results from similar researches will be used. Considering pain reduction of 1.5 units, variance of 1.8 with CI 95% and 80% power, and 10% dropouts, 135 participants were estimated to be suitable for this study.

### Statistical analysis

The comparison of the mean pain intensity of the three groups will be done using repeated measurement, and ANOVA in three different time intervals of before intervention, after the first intervention, and after the second intervention with repeated measures will be used to compare the mean scores of menstrual pain intensity between the groups at different time points. *P* value of less than 0.05 will be considered significant. The statistical analysis will be performed using SPSS version 22.

### Data management and monitoring

In order to optimize the completeness of data collection, researchers will enhance communication with subjects. Therefore, we will strengthen our connection with subjects through social media and phone to remind and monitor their medications. One of the researchers (FS) will be responsible for collecting data and will assign another individual for the task of data entry into the SPSS software. A data manager will check all the data without knowing the treatment allocation. This will be performed under supervision of SO and SK.

## Discussion

Persian yellow rose, with the scientific name *Rosa foetida*, is of the rose family and it is an edible flower as well. It is also a self-seeding, native flower in certain regions of Iran. The most important chemicals found in the rose family are vitamins, fennels, and flavonoids. Clinical studies on animals as well as human showed that fennels and flavonoids exert multiple biologic effects such as anti-microbial, anti-tumor, anti-pain, and anti-inflammatory effects [33, 34]. Moreover, studies reported that contextual factors related to primary dysmenorrhea self-care behaviors, along with self-management, can prove to be effective in pain reduction [30, 35–37]. This study will be the first clinical study on *Rosa foetida* along with self-care on dysmenorrhea. There are limited clinical researches on the effects of rose on dysmenorrhea and there are still questions regarding the effective complementary methods for reducing this bothering problem. Moreover, there is still no clinical study on the effectiveness of *Rosa foetida* on dysmenorrhea. Hence, if this study find a positive effect of *Rosa foetida* on dysmenorrhea, it can be suggested as an alternative therapy in dysmenorrhea.

### Trial status

Participant enrolment is progressing and data collection is going on. Participant recruitment started on October 2019 and is expected to end in March 2021.

## Data Availability

At the end of the study, a completely de-identified data set is scheduled to be delivered to an appropriate data archive.

## Abbreviations

CONSORT: Consolidated Standards of Reporting Trials
VAS: Visual analog scale
TENS: Transcutaneous electrical nerve stimulation
MDQ: Menstrual Distress Questionnaire
DSCS: Dysmenorrhea Self-Care Scale
IRCT: Iranian Clinical Trial Registration Center
